# Coat as a Dagger: The Use of Capsid Proteins to Perforate Membranes during Non-Enveloped DNA Viruses Trafficking

**DOI:** 10.3390/v6072899

**Published:** 2014-07-23

**Authors:** Eva Bilkova, Jitka Forstova, Levon Abrahamyan

**Affiliations:** Department of Genetics and Microbiology, Faculty of Science, Charles University in Prague, Vinicna 5, 12844, Prague 2, Czech Republic; E-Mails: bilkove@natur.cuni.cz (E.B.); jitka.forstova@natur.cuni.cz (J.F.)

**Keywords:** viruses, membrane perforation, viroporins, coat protein, capsid protein, polyomavirus, adenovirus, papillomavirus, parvovirus, cell entry, trafficking, endosome escape

## Abstract

To get access to the replication site, small non-enveloped DNA viruses have to cross the cell membrane using a limited number of capsid proteins, which also protect the viral genome in the extracellular environment. Most of DNA viruses have to reach the nucleus to replicate. The capsid proteins involved in transmembrane penetration are exposed or released during endosomal trafficking of the virus. Subsequently, the conserved domains of capsid proteins interact with cellular membranes and ensure their efficient permeabilization. This review summarizes our current knowledge concerning the role of capsid proteins of small non-enveloped DNA viruses in intracellular membrane perturbation in the early stages of infection.

## 1. Introduction

Viruses are cellular parasites that usurp the host cell energy and mechanisms for their own propagation. The first barrier they have to overcome at the cellular level is the plasmatic membrane. Enveloped viruses possess the phospholipid bilayer, which is often used to fuse with the host cell membrane. Subsequently, the viral capsid is released into the cytoplasm. Non-enveloped DNA viruses lacking the membrane jacket cannot use this principle. Instead, the protein coat enables their transmembrane penetration and ensures the next steps of the viral genome delivery to the site of amplification. Small DNA viruses, with the capsid diameter <100 nm, have only a limited number of capsid proteins to achieve this goal. Therefore, the capsids of small non-enveloped DNA viruses can be described as very efficient gene vectors. Understanding this step of infection, which is often rate-limiting, will help to design efficient gene vectors, applicable to various medical purposes. 

In general, the process of crossing the membrane by non-enveloped viruses has not been fully elucidated, even though it is a critical step of viral infection. The current knowledge concerning the role of small non-enveloped DNA virus capsid proteins in overcoming the membrane barrier during early phases of infection is the subject of this review. We compared four viral families described in detail in this paper—*Adenoviridae*, *Papillomaviridae*, *Polyomaviridae*, and *Parvoviridae*. These viruses share similarities in their “design”, and thus they overcome the same obstacles using similar weapons. It is beyond the scope of this review to cover the entire virus trafficking and uncoating picture, which also includes receptor binding, endocytosis and nuclear import. Instead, we focused on the mechanism of membrane penetration, the role of capsid proteins and conformational changes of the virion involved in this process, and recent advances in this field. The reviews that address the questions concerning trafficking of these viruses in a broader manner have been published elsewhere [1,2,3].

## 2. The Role of Capsid Proteins in Membrane Penetration

### 2.1. Adenoviridae

Members of the *Adenoviridae* family are non-enveloped viruses that infect vertebrates. Their genome consists of double-stranded linear DNA approximately 36 kbp long packaged into a capsid of about 90 nm in diameter. Human adenoviruses are divided into seven species, A to G, and are causative agents of multiple diseases, predominantly including respiratory infections, gastroenteritis or conjunctivitis.

The adenoviral capsid is a particle with icosahedral symmetry with fibers protruding from each of the 12 vertexes. Fibers are linked to the capsid via penton base. The most abundant protein of the capsid is hexon, represented in approximately 720 copies per virion [4], assembled in 240 trimers. The mature virions of adenoviruses also contain additional minor structural proteins IIIa, VI, VIII and IX. Other proteins and the viral genome are located inside the capsid. The proteins V, VII and µ bind the viral genome, and terminal proteins are covalently linked to its 5' ends. Cysteine protease L2/p23 is also located in the interior of the virion.

#### 2.1.1. Receptor Binding

The entry of subgroup C adenoviruses to the interior of the cell is initiated by binding of the fiber knob to the coxsackie-adenoviral receptor (CAR) on the cell surface, followed by interaction of the penton base RGD motifs with cellular α5 integrins [5,6,7,8]. While the main route of endocytosis of virions is via clathrin-coated pits, for some of the species, the macropinocytosis pathway has been reported [9,10,11,12,13,14].

Uncoating of the virus particle is necessary for successful infection, and events leading to membrane penetration already start at the cytoplasmic membrane. Initially, the interactions of viral particles with receptors and co-receptors support release of the fiber [15]. The importance of fiber dissociation was demonstrated by Nakano *et al.* [16]. The authors showed that fiber release is essential, but not sufficient for efficient cytosolic translocation of the virus. Moreover, their results demonstrated that fiber shedding and endocytosis are independent events [16]. This is consistent with the observation that deletion of RGD penton base motifs (which bind cell surface integrins as viral entry co-receptors) severely reduced internalization, but did not affect viral attachment [17,18]. 

These facts lead to the model of fibers being responsible for attachment, while internalization is the competence of the penton base protein. Therefore, the fiber protein can be deployed after it provides attachment, and this step is a prerequisite for endosomal escape. In addition, adenoviral fibers serve not only for virus attachment, but also as a timer determining the endosomal escape of the particle. This was demonstrated by experiments of Miyazawa *et al.* [19,20]. Adenovirus type 5 (Ad5, subgroup C) rapidly translocates to the nucleus within one hour. At the same time, Adenovirus type 7 (Ad7, subgroup B) virions are still clustered in membranous organelles of the cytoplasm. Trafficking kinetics of Ad5f7 chimeric vector, possessing Ad5 capsid and Ad7 fibers, resembles more Ad7 than Ad5 [19,20]. These experiments underline the importance of the fibers during very early phases of infection that precede cytosolic translocation. 

Furthermore, fiber shedding supports release of protein VI, one of the cement proteins straightening the capsid [15]. This protein plays a principal, but not an independent role in endosomal escape (see Sections below, 2.1.2.1).

#### 2.1.2. Endosome Escape

**Figure 1:**
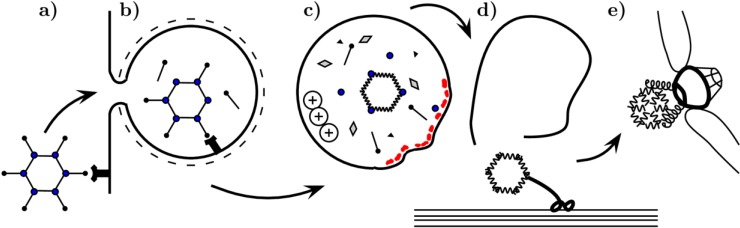
Schematic summary of early stages of adenoviral infection. Fibers interact with CAR receptor (**a**). The cell internalizes the virus via clathrin-coated pit (**b**). The endosomal interior becomes acidic: the viral particle undergoes conformational changes leading to disassembly. Fibers are shed, part of the penton base protein population and other proteins, including protein VI (red), are released (**c**). Protein VI induces vesicle rupture. The partially disassembled viral particle uses microtubules to reach nuclear proximity (**d**) and docks to the nuclear pore complex (**e**), where further disassembly allows the viral genome associated with proteins to reach the nuclear interior.

Capsid proteins act in a precisely concerted manner to allow the viral genome to escape from the endosome and subsequently reach the nucleus. As mentioned in the previous section, the first player is the fiber, which is released from the capsid. This is followed by release of other capsid proteins, mainly capsid-stabilizing proteins, IX and VI, penton base, and hexons [21,22]. The viral particle then penetrates the endosomal membrane and escapes to the cytosol. This step provides both a rescue from degradation by lysosomal enzymes and a way to enter the cell nucleus [23,24](Figure 1). In majority of the cases, endosome escape is a rapid and efficient process. Depending on the cell type, most adenoviruses need only 15 minutes post-infection or less to appear in the cytoplasm, except for subgroup B adenoviral particles, which can be found in the endosomal-lysosomal compartment up to eight hours post-infection [19,20,22,25,26]. 

The escape of adenovirus from an endocytic vesicle was observed using electron microscopy more than 50 years ago [27], but its precise mechanism remains to be determined. The relationship between adenoviral release and endosomal pH is rather controversial. Previously, the ability of adenoviral particle to permeabilize endosomes has been shown to require low pH [28,29]. Seth drew this conclusion from the experiment where membrane vesicles were exposed to adenovirus in buffers with differing pH [28]. Results of later publications came from the model using isolated endosomes [29]. In contrast, recent experiments using different inhibitors of endosomal acidification have shown that adenoviral penetration occurred efficiently even if pH of the endosomes was neutralized [30]. Later experiments have followed infection in single cells, a model providing better accuracy. Consistently, it was shown that the predominant site of endosome permeabilization and protein VI exposure is the proximity of the cell surface rather than early or late endosomes [25]. Thus, adenoviruses seem to escape from the vesicles that have not yet been acidified. It has been suggested that low pH at the plasma membrane might be sufficient to induce viral permeabilization of the endosomal membrane [30]. By way of explanation, adenoviral infection may need low pH, but the environment of the plasmatic membrane-derived vesicles may be sufficient. Observations of the pH dependence of viral inocula [28] would be consistent with this idea. Nevertheless, this hypothesis would need to be validated. Both the pH of the viral inoculum and the acidity of culture media might be involved. Hence, the differences in observations might reflect variations between cell lines or experimental settings. 

Permeabilization of endocytic vesicles occurs in rather a massive and catastrophic manner—as a membrane rupture. This is supported by the experiment describing adenoviral infection allowing release from vesicles of such large objects as whole parvoviruses or 70 kDa dextran [29,31]. Parvoviruses (size 18–26 nm) deficient in endosomal escape could perform successful infection when co-infected with Ad5 or upon vesicle lysis induced by polyethylenimine [31]. The adenoviral infection was shown to induce release of up to 46% of the internalized biotin-dextran molecules from isolated endosomes without size selectivity [29].

Successful penetration requires proper function of several capsid proteins and the right length of the viral genome packed in the virion. Therefore, an adenoviral chimeric particle containing short 12.6 kb DNA (instead of the 36 kb genome) has abnormal protein composition, lacks capsid-stabilizing protein IX, and cannot efficiently escape endosomes [17]. Delivery of the viral genome to the nucleus also requires completely processed pre-terminal protein [32].

Another protein involved in correct uncoating and successful penetration is penton base. Similarly as the fiber protein, it plays a role in the first steps of the infection. Part of the population of penton base protein is lost during the time that virus spends in the endosome [33]. In view of the viral vesicle escape, it has been shown initially that application of anti-penton base serum inhibited rupture of vesicles derived from the cytoplasmic membrane [28]. Later, another report revealed that RGD motifs of penton base (which interacts with viral co-receptors) facilitate endosome escape [18]. These observations may be related to penton base release, which can probably be blocked by antibodies and favored by binding of co-receptors. Furthermore, dissociation of penton base may be required for the release of protein VI, which is located in the capsid interior and has a principal role in endosome escape (described in the next section). It is also possible that protein VI exits the particle only after dissociation of vertex proteins, including penton base.

In contrast, the involvement of adenoviral protease L3/p23 in endosome escape is rather debatable. An early report described a virus with the protease inactivated by reducing and alkylating agents. Under these conditions, virus with alkylated viral protease was able to reach the cytosol [34]. A recent study has identified cleavage of several capsid proteins by adenoviral protease, including the N-terminus of protein VI, which is crucial for endosome escape [4]. In theory, this peptide could be released upon endocytosis and disassemble from the particle, and could mediate membrane destabilization in a protease-dependent manner. Nevertheless, currently there is only indirect evidence of this concept, which is contradictory to earlier observations.

##### 2.1.2.1. Role of Protein VI

Several experiments demonstrated the ability of protein VI to induce membrane lysis and its principal role in endosome rupture, crucial for successful infection. Protein VI (22 kDa) is one of the proteins released from adenoviral capsid during disassembly within endosomes. It is expressed as a 250 amino acid long pre-protein. The mature protein is generated by adenoviral protease cleavage at both N- and C-terminus, generating a 206 amino acid long protein [35]. Each capsid has approximately 360 copies of the protein per virion [4,36,37]. According to analysis of cryo-electron microscopy structures of adenoviral capsids, it is thought that protein VI is located in the interior cavity of the hexon inside the capsid [38,39,40]. 

Protein VI has multiple roles in infection. In the late stages, it facilitates particle assembly and maturation. It was shown that protein VI also mediates nuclear import of the hexons to the nucleus [35], and its C-terminus functions as a cofactor of adenoviral protease [41]. Furthermore, in early phases of infection, but after endosomal escape, this coat protein also facilitates microtubule-dependent trafficking of the virus towards the nucleus [42].

As was shown by Wiethoff *et al.*, lysis of the endosome requires the N-terminal domain of protein VI, which forms amphipathic α-helix and is highly conserved among adenoviral species [33]. Research by this group has shown that the membrane disruption ability of protein VI is possessed by both the precursor and mature form of the protein and that this activity is pH independent [33]. Later experiments confirmed the protein’s role in infection and identified lysine 40 as an amino acid critical for the lytic activity during the infection. Consequently, substitution of this residue decreases the level of protein VI insertion into membranes and inhibits virus lysis of endosomal membranes [43]. 

Another mutation in the α-helical domain of protein VI, substitution of glycine in position 48 by cysteine (G48→C), was also shown to lower its membrane disruption activity. This substitution resulted in formation of aberrant disulfide bonds creating dimers of protein VI within the adenoviral particle. In addition, the release of the mutated protein VI in endosomes was more restricted and the mutation impaired the protein ability to lyse liposomes [44].

Additionally, Maier *et al.* [45] demonstrated the ability of N-terminal α-helix to induce membrane curvature and membrane fragmentation. *In vitro* studies using a peptide corresponding to this part of protein VI showed that it inserts itself into the membrane nearly in parallel to the membrane surface. Data suggested a model in which binding of protein VI would induce positive membrane curvature responsible for large-scale membrane disruption. This means that the lipid bilayer would bend in a convex manner before its rupture [45].

Thus, the overall evidence suggests that the release of protein VI is indispensable for the infection and possible only from a partially disassembled particle. It is not surprising that one of the host-cell defense strategies is to impede the exposure of the N-terminal domain of viral protein VI. This host antiviral mechanism involves defensins, which belong to the family of antimicrobial peptides abundant in cells and tissues [46]. Defensins do that by stabilizing the adenovirus capsid and inhibiting the release of protein VI [47]. Moreover, several groups also demonstrated that defensins bind the vertex complex and fiber [47,48,49,50]. 

Molecules of the protein VI of Ad5, released in the endosome, remain associated with the vesicle membrane after the escape of endosome [25]. Nevertheless, the release of protein VI is not complete. Part of the protein VI population stays bound to the capsid and plays a role in the virus movement towards the nucleus [42].

The overall data suggest a model in which successful fiber release and shedding of the penton base as a result of interaction with the receptor and co-receptor, and, possibly, acidic environment, create access to the capsid interior and allow escape of the protein VI located beneath hexons. This protein interacts with the lipid bilayer, induces lysis by membrane curvature and the disassembled virus then escapes to the cytosol. 

#### 2.1.3. Nuclear Translocation

After reaching the cytosol, adenovirus particles travel closer to the nucleus. This can be evidenced by detecting the viral capsids near the centrosome. In order to reach the nucleus, adenoviruses use microtubules and their motors [51,52]. Next, particles dock by the nuclear pore complex and undergo further disassembly through the interactions with host proteins such as chaperone Hsc70 [53], histone H1, and importins [54]. Finally, the viral protein VII and the host nuclear import and export factors promote translocation of viral DNA into the nucleus [54,55,56].

### 2.2. Parvoviridae

Parvoviruses represent the smallest known DNA virus family. Viruses of the *Parvoviridae* family encompass genera infecting vertebrates as well as insects. Genus *Erythrovirus* includes human pathogen B19, an agent causing erythema infectiosum (fifth disease). The parvoviral genome is a single-stranded DNA of approximately 5 kbp, flanked with palindrome ends forming double-strand T or Y shape structures. Their icosahedral, non-enveloped capsid ranging between 18 and 26 nm in diameter is composed of sixty copies of structural proteins. Of these proteins, VP1 (82 kDa) represents approximately 10% of the capsid proteins. It comprises the entire sequence of VP2 (60 kDa) coat protein and a unique N-terminal region (VP1u). VP2 protein is the major capsid component for most parvoviruses, constituting over 90% of the capsid proteins [57,58,59,60]. In contrast, adeno-associated viruses (AAVs) use VP3 as a major coat protein, which represents about 85% of total capsid proteins [61]. This protein is generated by N-terminal cleavage of VP2 by host cell proteases.

#### 2.2.1. Cell Entry

Parvoviruses use receptor-mediated endocytosis to enter host cells. The list of receptors and attachment molecules used by parvovirus genera is long and diverse. For example, while adeno-associated virus, serotype 2 (AAV2), uses heparin sulfate as a receptor [62,63], the canine and feline parvoviruses (CPV and FPV, respectively) interact with transferrin receptor [64,65]. Attachment of Bovine parvovirus (BPV) occurs via sialic acid binding [66]. Another parvovirus, the human parvovirus B19, was shown to use P antigen as a receptor [67], and Ku80 autoantigen [68] and α5β1 integrin molecules as co-receptors [69]. Overall, the clathrin-coated pits are the major, but not the exclusive, mediators of parvovirus internalization [62,70,71,72,73,74,75]. 

**Figure 2:**
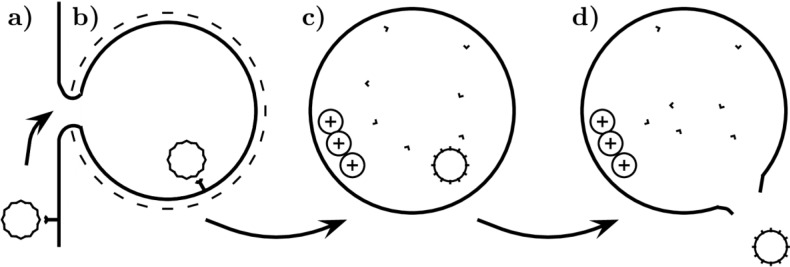
Scheme of early stages of parvovirus infection. The parvovirus particle interacts with a receptor and becomes internalized using clathrin-coated pit (**a**,**b**), where pH decreases (+, protons) and capsid proteins undergo conformational changes, N-terminus of VP2 may be cleaved, N-terminus of VP1 is exposed on the capsid surface (**c**). The parvovirus leaves the endosome by a yet undefined mechanism (**d**).

In the later stages of infection, parvoviral capsids can be found in several cell compartments, predominantly those of endosomal origin. In contrast, AAV5 represents one of the few exceptions among DNA viruses since its capsids accumulate in the Golgi apparatus [72]. After endocytosis, the virus moves towards the nucleus and accumulates in the perinuclear area. Their preferred locations are late endosomes and lysosomes [71,75,76,77]. Finally, successful infection requires intact microtubules and is pH-dependent, suggesting that the passage through one of the acidic endosomal vesicles is vital for conformational changes preceding the endosome escape [62,71,73,75,76,78,79,80]. The early phase of infection preceding endosome escape is summarized in Figure 2.

#### 2.2.2. Endosome Escape and Capsid Disassembly

Incubation of capsids in acidic endosomes induces conformational changes of capsid proteins that lead to viral endosome egress. The current knowledge supports the model where capsids released to the cytosol undergo further disassembly and continue their mission to reach the nucleus. 

Viral capsids are greatly responsive to the vesicle internal environment. For example, it was shown that the sphingomyelin and phosphatidylserine bilayer induces structural changes of CPV capsids, which seem to differ from the conformational alteration caused by low pH incubation [81]. Another potent inducer of structural transitions is the change of pH. The sensitivity to pH is a dynamic process and this was demonstrated for AAV2 and CPV infection. While AAV2 infection was sensitive to the rising endosomal pH during the initial 30 minutes [62], CPV infection was sensitive up to 90 minutes post infection (p.i.) [71]. Moreover, the low pH facilitates the exposure of VP1 N-termini, which are buried inside the capsid. This transition and its importance for infection were well documented for several parvovirus species [82,83,84,85]. Additionally, it was reported that incubation in urea (3 to 5 M) or elevated temperature (around 50 °C, depending on the viral species) are also sufficient for this VP1u exposure [86,87,88,89,90]. The N-terminus of the VP1 polypeptide likely protrudes on the surface of the virion through the capsid fivefold cylinder [86,91,92]. Importantly, the N-terminus of the capsid protein possesses motifs vital for endosomal escape (described further below). The exposure of the VP1 specific domains can also be enhanced by proteolysis of the VP2 N-terminus, which also occurs after internalization [82,86]. The results suggest that parvovirus needs low pH as an inducer of conformational changes of the capsid proteins. The low pH also ensures optimal conditions for the activity of endosomal enzymes, crucial for correct capsid disassembly. 

Endosomal proteases may play an important role in uncoating parvoviruses. Specifically, it was shown that cysteine endosomal proteases, cathepsin B, and L, interact with both AAV2 and AAV8 capsids, and cleave the coat proteins. On the other hand, AAV5 seems not to require these enzymes for infection [93].

Low pH, endosomal proteases, and interaction with membrane phospholipids may contribute in concert to conformational changes of the viral capsids. The most important, but not the only one for successful endosomal escape, seems to be exposure of the unique region of VP1 [84]. 

Parvovirus infection, unlike adenovirus infection, causes rather small perforations than a massive rupture in the membrane. CPV infection allows cytosolic translocation of 3 kDa, but not 10 kDa dextran [84]. In contrast to CPV, Adenovirus type 1 permeabilizes endosomes for alpha-sarcin cytosolic translocation (molecular weight approximately 20 kDa, diameter 5 nm) [71]. These data suggest that the parvoviral capsid induces small perforations, which cannot allow large-scale translocation of soluble complexes.

##### 2.2.2.1. VP1 Unique Region

Protrusion of the N-terminus of VP1 is indispensable for delivery of the genome from the perinuclear endosomal compartments to the nucleus. This part of the major capsid protein possesses a basic amino acid region and conserved domain with phospholipase A2 (PLA_2_) activity (which is Ca^2+^ dependent, Figure 2) [94,95,96,97].

Phospholipases hydrolyze phospholipid esters at the sn-2 position to fatty acids and lysophospholipids. One of the prominent products of this reaction is arachidonic acid, which is used for synthesis of eicosanoids, prostaglandins, and leukotrienes that have an important role in inflammatory processes.

Indeed, arachidonic acid can be found in cell culture medium infected with porcine parvovirus. PLA_2_ enzyme activity differs among parvoviral species. Porcine parvoviral PLA_2_ displays approximately 10^3^ times higher activity than those of AAV2 and B19 enzymes. Parvoviral PLAs are not very specific and hydrolyze all main classes of glycerophospholipids with the exception of phosphatidylinositol [98]. In addition, it was shown that human fibroblast-like synoviocytes (HFLSs) incubated with the bacterially-expressed unique region of the VP1 protein (VP1u) increased production of prostaglandin E2 and cyclooxygenase [99]. These metabolites are involved in pathophysiological processes such as inflammation and tissue damage [100,101].

**Figure 3:**
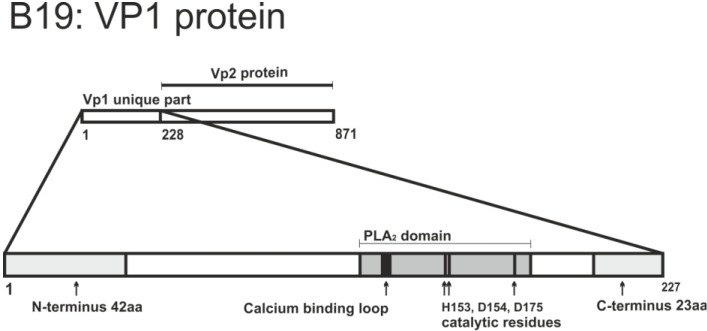
Overview of sequence features of B19 VP1 unique region.

The phospholipase A2 (PLA_2_) activity of the major capsid protein plays an essential role in the parvoviruses escape from endosomes. If point mutations are introduced in the PLA_2_ active center, the infectivity of MVM, AAV2 and B19 is severely impaired [31,97,102,103] (see Figure 3 for details concerning B19). These mutations do not influence either capsid assembly, or cell binding and entry. However, the viral genomes of these mutants are not delivered to the nucleus, and are found in the perinuclear vesicles. Furthermore, mutated viruses show delayed onset and reduced amount of early gene expression. Interestingly, the MVM PLA_2_ mutant ability to reach the nucleus can be rescued by co-infection with the wild type virus, endosome lysis by polyethylenimine, or co-infection with adenovirus, which induces endosome membrane rupture (see Section 2.1.2 for details) [31,97,102,103]. Thus, endoosmolytic activity of adenoviruses appeared to be involved in complementation of PLA_2_ mutants [31,97,102,103]. The importance of PLA_2_ for the parvovirus life cycle was further proved by the observation that treatment with PLA_2_ inhibitors abolished infection of CPV [84]. Altogether, these data strongly support the model of PLA_2_ having a principal role in penetration of parvovirus particles into the cytosol. 

In addition to the PLA_2_ domain *per se*, the amino acid residues outside the PLA_2_, also affect its activity. Almost all polypeptide parts of the VP1 unique region are involved in enzymatic activity; the very N- and C-termini being the most important. For instance, deletion of 42 amino acids from the N-terminus or 23 amino acids from the C-terminus decreases PLA_2_ activity by 80% or more [104] (Figure 3 and Table 1). 

**Table 1:** Overview of sequence features of B19 VP1 unique region residues and motifs involved in endosomal escape. The biological effects of mutations are included if relevant.

Residues and motifs	Biological properties	Introduced mutation	Effects of mutation	Reference
123–181	PLA2 motif, based on sequence alignment and homology	Fragment of VP1 from 2 to 240 aa	Protein exhibits PLA2 activity	[97]
131–227	includes PLA2 motif	Deletion 131-227	Abolished PLA2 activity of protein	[99]
H153 (histidine) D175 (aspartic acid)	Catalytic residues	H153A, D175A point substitution	Abolished PLA2 activity of protein	[99]
P133 (proline)	Calcium binding loop residue	P133R substitution	Abolished PLA2 activity of protein	[99]
1–42 (N-terminus 42 aa) 205–227 (C-terminus 23 aa)	Outside PLA2 motif but required for its function	Deletion 43–227, deletion 1–204	Severely impaired PLA2 activity of protein	[104]
E176 (glutamic acid)	Proximal to catalytic residues of PLA2 motif	E176D substitution	Abolished PLA2 activity of protein and infection	[103]

*In vitro* experiments revealed that CPV capsids disrupt artificial membranes in low pH conditions [105]. The fluidizing effect was also observed in the endosomal membranes. Authors assigned this function to the unique region of VP1. The finding is supported by the observation that capsids composed solely of VP2 did not exhibit any effect on intracellular membranes. Nevertheless, the VP1 protein alone was not sufficient for this activity as well. On the other hand, Deng *et al.* [104] showed that treatment of cells with the VP1u protein resulted in significant changes in cell morphology, accompanied by massive fatty acid release. This observations suggest that the membrane integrity in cells treated with VP1u was dramatically decreased [104].

The ability of the PLA_2_ domain to manipulate membranes was also demonstrated by interaction of B19 VP1 with red blood cells (RBC). It was shown that a proportion of the capsids externalize the VP1 unique region upon binding with RBC. Furthermore, *in vitro* RBC exposure to the B19 virus did not induce hemolysis, but the integrity of the cell membrane was impaired because of increased osmotic fragility of B19-exposed RBC, as measured by incubation in hypotonic buffer. Moreover, this effect on cellular membranes was attributed to PLA_2_ activity of the exposed VP1u [106]. Therefore, it is likely that parvoviruses use the enzymatic activity of PLA_2_ to reach the cytoplasm.

Several reports revealed that the phospholipase activity of VP1 affected the cell signaling during viral infection. The PLA_2_ activity was shown to induce entry of Ca^2+^ ions into the cytoplasm via calcium release-activated calcium current (I_CRAC_ channel) [107]. The concentration of Ca^2+^ ions is a sensitive modulator of many signaling pathways, and parvoviral infection influences one or more of them via PLA_2_ activity. In addition, injection of RNA encoding B19 VP1 to *Xenopus* oocytes was shown to inhibit host cell Na^+^/K^+^ ATPase, while PLA_2_ negative mutant variant (substitution in catalytic site H153→A) failed to do so. This effect can be inhibited by treatment with PLA_2_-specific inhibitor or mimicked by lysophosphatidylcholine, a PLA_2_ activity product [108]. Furthermore, UT7-Epo cells (human leukemic cell line capable of growing in erythropoietin, permissive to B19 infection) exposed to the VP1u protein exhibited activated TNFα and NFκB gene expression [104]. These effects are likely to contribute to the pathophysiology of parvovirus B19 infection.

Unfortunately, the sequence of conformational changes in the capsid and the function of VP1u are not well understood. A recent study by Venkatakrishnan *et al.* (2013), in which computational methods, circular dichroism and electron microscopy were used, shed some light on this topic. Their results suggested that the unique region of the VP1 protein of AAVs has an α-helical structure, while the common VP1/VP2 region is likely to be disordered. Moreover, the VP1 unique region seems to lose the folded structure upon incubation of the virus particles in acidic environment [109]. This conformational change can provide the flexibility needed for externalization of VP1u in acidic endosomes, which is essential for infection (Table 1 and Table 3). Interestingly, the low pH and refolded state may allow externalization of the N-terminus of VP1 protein, but are not favorable for PLA_2_ enzymatic activity required for infection. The optimum pH for parvovirus PLA_2_ activity is around 6–7 and the activity decreases rapidly with further pH decrease [98]. On the other hand, a partially active enzyme could be sufficient for membrane permeabilization, since pH 5 does not block PLA_2_ enzymatic activity completely. Alternatively, interaction with the lipid membrane may support adoption of the properly folded state of the enzyme.

Despite the number of studies targeted at explaining the role of PLA_2_ in parvovirus infection and the identification of the host cell processes involved in virus endosomal escape, several questions remain open. It is plausible that hydrolysis of the membrane components such as phospholipids helps to induce the membrane curvature and fluidizing. These changes may provide virus with the endosome escape pathway through creation of small perforations in the endosomal membrane. Virus-mediated interference with intracellular ion concentrations and modulation of gene expression may also be involved. However, it is more probable that these processes influence later steps of infection, e.g., translocation through the nuclear envelope or viral assembly and release.

#### 2.2.3. Further Trafficking and Entry into the Nucleus

Parvovirus particles seem to escape from membranous vesicles directly to the cytoplasm, where they interact with host cytosolic factors. The model including the particle passage through the cytoplasm is supported by the report showing that anti-CPV antibodies effectively inhibit infection when introduced into the cytoplasm [78].

The next challenge for the parvovirus genome is to overcome the cell nuclear envelope. The CPV capsids are mainly localized in the vicinity of nuclei 30–90 min p.i., but viral genomes trafficked into the nucleus several hours later. When capsids were injected directly into the cytoplasm, they rapidly translocated near the nuclei, but entered the nuclear compartment three to six hours later [71,77]. Lux and co-authors [110] also described capsid nuclear translocation as an inefficient process.

In theory, the parvoviral capsid is small enough to pass the nuclear pore complex. However, studies of MVM (minute virus of mice) suggested that this parvovirus might use a different nuclear translocation mechanism. Initially, a study by Cohen *et al.* [111,112] revealed that MVM infection changed the nuclear morphology. Authors also observed breaks in the nuclear envelope. Further analysis showed that host cell caspases, but not viral PLA_2_, are involved in this process [111,112]. On the other hand, some results concerning AAV and recombinant AAV vector infection dependence on the nuclear pore complex are contradictory [113,114]. Thus, the manner by which parvoviruses achieve the nuclear envelope perforation remains unknown, as well as the precise mechanism of their nuclear translocation. 

### 2.3. Papillomaviridae

Papillomavirus virions have a diameter of about 55 nm and their protein coat comprises two structural proteins, L1 and L2. The major capsid protein, L1, present in 360 copies per capsid, assembles into 72 pentamers forming T = 7 icosahedral lattice [115]. Protein L2 occupies the interior cavity of the L1 pentamer. The papillomavirus genome is a circular double-stranded DNA of approximately 8 kbp.

Papillomaviruses infect basal keratinocytes, but viral progeny production requires differentiated keratinocyte cells. Human papillomaviruses can induce benign, self-limiting tumors of skin, and mucosa. These lesions may rarely progress to carcinomas of the cervix, vagina, penis, and other parts of the body.

#### 2.3.1. Cell Entry

Most papillomaviruses use heparan sulfate proteoglycan (HSPG) as a primary receptor [116,117]. After binding to the primary receptor, the particle undergoes several conformational changes. Interaction with HSPG induces exposure of the L2 N-terminus on the virion surface and a 17–36 aa long epitope named RG-1 becomes accessible to antibodies (Table 2). Antibodies against this epitope neutralized the virus and a vaccine based on this epitope induced protective immunity in mice [118,119,120].

At least in the case of HPV 16 and 18, this conformational change in L2 is facilitated by cyclophilin B isomerase (CyPB). It was shown that CyPB triggered exposure of the L2 N-terminus and that L2 proteolytic cleavage by cellular furin convertase are vital for effective delivery of the papillomavirus genome to the nucleus and infection [121]. Interestingly, after furin-mediated cleavage, the virus loses its affinity to the primary attachment receptor but gains affinity to the uptake receptor [122,123]. 

#### 2.3.2. Intracellular Trafficking

Papillomaviruses seem to use diverse mechanisms for entry and intracellular trafficking and there has been much debate in the literature and inconsistency among many studies. Papillomaviruses were described to use clathrin-coated pits and caveolae to enter cells [124,125,126,127] as well as a novel, non-clathrin, non-caveolin, tetraspanin-enriched microdomain dependent invasion route related to macropinocytosis [128,129,130]. Recently, the involvement of trans-Golgi network (TGN) in HPV infection was also shown [131]. Additionally, the presence of BPV1 and HPV16 in ER was reported, confirming the importance of TGN and ER in papillomavirus intracellular trafficking [132,133].

A number of papers described the presence of papillomavirus in the late endosomal and lysosomal compartments of the perinuclear region [127,129,130,134]. It is not clear whether this represents the productive infection pathway, since the majority of particles fail to deliver viral DNA to the nucleus [135] and L1 protein and genome are presumably degraded in the lysosome. Further studies are needed to clarify the intracellular trafficking steps of this viral family.

It is likely that papillomaviruses have to pass one of the acidic endosomal compartments because the rise in endosomal pH inhibits infection [129,136,137]. A recent study has demonstrated this dependence by showing that papillomavirus uncoating and infection is dependent on the activity of vacuolar ATPase, which drives acidification of endosomes [138]. Viral particles undergo important changes inside the endocytic vesicle. The majority of HPV16 L1 epitopes become unrecognizable by antibodies after internalization [139], while the viral genome becomes detectable [119]. This suggests that the virus needs acidic environment to induce uncoating and disassembly. Protein disulfide isomerases and cysteine proteinases are likely to provide part of uncoating, since they are required for infection [135,137].

#### 2.3.3. Endosome Escape

**Figure 4:**
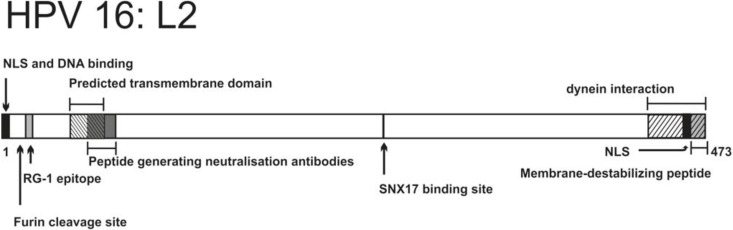
Schematic representation of HPV16 L2 structural and functional features (NLS—Nuclear Localization Signal).

Increasing evidence suggests that the L2 protein is involved in the viral genome escape from membranous vesicles, but the mechanism of L2-mediated escape has not yet been elucidated. Protein L2 is located in the interior cavity of the L1 pentamer via hydrophobic interactions, using its C-terminus [140]. According to Buck *et al.* [141], pseudovirions can have up to 72 copies of the L2 protein per particle, while an earlier report quoted less copies—about 12 per particle [142]. The majority of the L2 protein is hidden inside the capsid. Only a minor part of the protein (approximately 60 to 120 aa) is accessible from outside the capsid [143,144]. Proteins L2 of BPV-1 and HPV16 have nuclear localization signals (NLS) on both the N- and C-terminus [145,146,147]. Interestingly, the C-terminal NLS of L2 BPV1 also binds DNA [147], and the N-terminal NLS of L2 HPV16 overlaps with the DNA-binding region [148] (depicted in Figure 4).

It is believed that L2 protein plays several roles in the virus life cycle. This capsid protein facilitates viral DNA encapsidation during virion assembly and is involved in cell binding and internalization of the virus [149,150,151]. Furthermore, L2-deficient virions exhibit a drastic reduction in infectivity [152,153]. 

##### 2.3.3.1. L2 Sequence Properties Related to Endosome Escape

Several regions of the amino acid sequence of L2 have been proven to be involved in the endosome escape of papillomaviruses. One of the most intensively studied subjects is the L2 of HPV16. Its structural and functional features are summarized in Figure 4 and Table 2. HPV16 L2 was shown to be dispensable for viral uncoating but crucial for endosome egress [154]. As mentioned in the previous section, the N-terminus of L2, including the RG-1 epitope, is exposed and cleaved during virus entry, and this process is required for endosome escape and nuclear translocation of the virus [118,119,121]. This part of the protein also contains a conserved disulfide bond between cysteine residues C22 and C28. Thus, it is not surprising that disruption of the C22–C28 disulfide bond makes HPV16 virions unable to exit the endolysosomal compartment [118,155,156]. 

Bronnimann *et al.* [157] provided valuable insights into the role of the conserved L2 region within residues 45 to 67. They have shown that this region forms an α-helix in the lipid environment *in vitro*, and acts as a transmembrane domain. Introduction of point mutations into this domain abolished L2 transmembrane properties and inhibited infection, trapping viral DNA in endosomal compartments. Furthermore, the importance of the conserved GxxxG motif within this region and the tendency of the predicted TM domain to oligomerize via GxxxG motifs have been demonstrated [157]. Interestingly, serum against the epitope 56–75 aa of HPV16 L2 polypeptide efficiently neutralized infection by blocking entry and, more significantly, transport of viral genomes to the nucleus [144,158] (Table 2).

Additionally, Kamper *et al.* [154] revealed important membrane-lytic properties of the peptide representing the last 23 amino acids (approximately residues 445–470 aa, see Figure 4 and Table 2) at the C-terminus of the L2 protein of BPV1, HPV16, 18, and 33. Their study demonstrated strong membrane-disrupting activity of this peptide. The key insights were drawn from the experiments showing that the peptide lysed bacteria and eukaryotic cells more effectively at pH 6 than at neutral pH, which is consistent with the low endosomal pH requirement for infection. Of note, BPV1 pseudovirions harboring a truncated L2 C-terminus (without the last nine aa) were retained in the endosomal compartments. Therefore, it is highly plausible that the L2 C-terminus assists escape of viral genomes from the endosomes.

Unfortunately, the precise mechanism of endosomal escape mediated by the L2 protein remains elusive. It seems that the L2 protein also directs the virus from acidic endosomes to other membranous compartments (described in Section 2.3.3.2) and this redirecting is essential for productive infection. It was also shown that L2 protein has at least two domains with affinity to membranes (amino acids 45–67 and 451–473), which could span the membrane after partial capsid disassembly. This properties of L2 might support its membrane incorporation and help to form oligomers and generate the pore [157]. 

**Table 2:** Overview of HPV16 L2 protein residues and sequence motifs involved in the endosomal escape of the papillomavirus. The biological effects of mutations are included if relevant.

Residues and motifs	Biological properties	Introduced mutation	Effects of mutation	Reference
17–34 (RG-1 epitope)	Anti RG-1 antibodies have neutralizing properties Epitope exposed during early stages of infection	Not relevant	Not relevant	[118,119,120]
9–12 (RRKR)	Furin cleavage site	R12S substitution	Impaired infectivity, virus retained in endosomal vesicles	[119]
C22, C28 (cysteines)	Form a disulfide bond	C22S and/or C28S substitution	Virions lack infectivity, internalization and trafficking to endolysosomes are not affected	[155,156]
45–67	Predicted transmembrane domain, adopts α-helical structure	e.g., G56V, G57V substitution, A55, A60 insertion	Non-infectious virions, viral DNA trapped in an endosomal compartment during infection	[157]
56–75	Antipeptide serum inhibits infection, mainly by blocking the viral genome transport to the nucleus	Not relevant	Not relevant	[158]
254–257	Interaction with SNX17	N254A substitution	Abolished infectivity, capsid targeting to lysosomes increased	[159]
451–473 (last 23aa at C-terminus)	Membrane-destabilizing peptide	Deletion 455–473 or 465–473	Abolished infectivity, viral genome retained in endosomes during infection	[154]

##### 2.3.3.2. L2 and Viral Intracellular Targeting

The results of several studies indicate that L2 helps the papillomavirus to exit from the endolysosomal compartment to the Golgi and/or ER (the destination probably depends on the species) (Table 3). This endosome escape is crucial for infection, likely avoiding sorting of the virus to the lysosomal degradation. Presumably, this event precedes transmembrane translocation.

An increasing number of reports show that the L2 protein interacts with numerous host proteins involved in cellular trafficking. For example, Marusic *et al.* [159] described interaction of HPV16 L2 with sorting nexin 17 (SNX17), a protein involved in endosome recycling. Later, the interaction of SNX17 with several papillomaviruses has also been shown (including HPV11, BPV-1, HPV5) [160]. Furthermore, infection of all tested papillomavirus species was significantly reduced in SNX17-silenced cells. Therefore, SNX17 binding by the L2 protein seems to be conserved across the papillomavirus family. The results of Marusic *et al.* suggested that SNX17 may help retain the particles in the endosomal compartments and prevent their lysosomal degradation; consequently, SNX17 may assist the virus in early trafficking steps.

**Table 3:** Comparison of features of membrane penetration capsid proteins (MPCP) relevant for transmembrane translocation of non-enveloped DNA viruses during early stages of infection. Some features are based on the current models and proposed mechanisms described in the text.

Features	Adenoviridae	Parvoviridae	Papillomaviridae	Polyomaviridae
**MPCP**	VI	VP1	L2	VP2, VP3
**Weight of MPCP (relative to the capsid)**	5%	10%	12%	14%
**MPCP exposed on the surface** **(>15%)**	no	No	no	no
**Site of viral genome escape from the vesicle**	endosome	Endosome	Trans-Golgi network? ER?	ER
**Conditions required for MPCP exposure**	Partial capsid disassembly, release of other capsid proteins	High temperature, low pH, cleavage of other capsid proteins	Interaction of the capsid with primary receptor, extracellular enzymes	ER resident enzymes, interaction with the membrane, low pH for MPyV
**MPCP associates with the capsid during membrane damage**	no	Yes	no (but associates with the genome)	not known
**MPCP forms oligomers**	not determined	not determined	yes	yes

Other interaction partners of papillomaviruses involved in vesicular trafficking that might be involved in further steps of infection have been reported. For instance, the L2 protein of BPV1 was shown to bind syntaxin 18 (STX18), a tSNARE protein, which plays an important role in vesicular transport between the ER and Golgi, through amino acids 40 to 44 [132,161]. Flag-tagged STX18 expression inhibited both trafficking to ER and BPV1 pseudovirion ability to reach the perinuclear region and to propagate. Additionally, the transport protein particle complex subunit 8 (TRAPPC8), which seems to be involved in the same trafficking pathway, interacts with L2 of HPV51 (strain Ma), HPV16, and HPV31. The question emerges how direct interaction with an anterograde trafficking component would help papillomavirus infection. Authors suggested that binding of L2 to TRAPPC8 could impair its function and result in Golgi destabilization, which might be helpful for genome escape [162]. Concepts applicable to TRAPPC8 may also apply to the L2 binding of syntaxin 18. These interactions indicate L2 as an important factor in intracellular papillomavirus trafficking, but the context and precise purpose of these interactions remain to be defined. 

In a more general manner, experiments of Lipovsky *et al.* demonstrated that retrograde transport factors are required for papillomavirus infection and that HPV proteins interact with the retromer complex to reach the trans-Golgi network [131]. Currently, it seems to be widely accepted that papillomaviruses use microtubules to travel towards the nuclear proximity [127,136,163,164]. In addition, the L2 protein of HPV16 and HPV33 was shown to interact with dynein via a conserved C-terminal 40 aa motif [165,166]. It was suggested that dynein is required for the nuclear translocation of the L2/DNA complex, but the possibility that dynein can be involved in intracellular endosomal transport of internalized viruses has not been excluded. 

To sum up these findings, one theory proposes that the N-terminus and C-terminus of L2 are exposed during virus disassembly and interact with the membrane, thanks to their hydrophobic sequences. Viruses, by spanning the membrane and in order to reach one of the perinuclear compartments, recruit several transport factors including the endosome recycling and retrograde transport ones, and, thereafter, they escape lysosomal degradation [131]. Therefore, it seems that one of the roles of the minor L2 coat protein in cytosolic translocation is to assist virus delivery from acidic endosomes to the trans-Golgi/Golgi and possibly to ER network, where membrane permeabilization of the virus can occur. Since the viral genome reaches the nucleus in the complex with L2 [167] and this coat protein has domains able to disrupt membranes, it is likely that L2 also promotes membrane penetration by a yet undefined mechanism (described and discussed in the above Section 2.3.3.1).

#### 2.3.4. Further Trafficking and Possible Membrane Penetration Site

While L2 accompanies the viral genome to the nucleus, the L1 viral protein seems to be degraded [135,167,168]. Recent experiments suggested [131] that the virus enters the ER or Golgi compartment, avoiding lysosomal degradation (Table 3). Next, the viral DNA complex with L2 may escape to the cytosol and translocate through the nuclear pore. 

Another possible scenario is the nuclear membrane penetration (in a similar way as parvoviruses, see Section 2.2.3) either from ER/Golgi or from the cytoplasm by an unknown mechanism, which is likely to use the L2 protein, since it stays in complex with the genome until it reaches the nucleus. For this purpose, the L2 protein possesses several hydrophobic domains that can support protein integration into the membrane (Table 4). For example, the C-terminus domains of L2 possess the membrane disruption activity and could be involved in membrane permeabilization. It was reported that multimerization of L2 may create a pore in the nuclear envelope and help transmembrane translocation of the viral genome. Interestingly, a peptide of the L1 protein can also permeabilize cells [169], leaving an opportunity to speculate that the minor part of the non-degraded population of L1 protein could also help membrane permeabilization.

In conclusion, we can say that L2 has a vital role in viral genome transfer from the endolysosomal compartment to the nucleus. However, the precise mechanism by which papillomaviruses manage to do this is still under the debate. For instance, definitive evidence of the presence of viral DNA in the cytoplasm is lacking, and reports of the presence of free viral particles there are exceptional [158]. Thus, our knowledge concerning papillomavirus nuclear translocation is very limited; use of the nuclear pore complex to enter the nucleus has not been proved. Much more needs to be done to further our understanding of these steps of papillomavirus infection.

### 2.4. Polyomaviridae

Polyomaviruses are small, non-enveloped viruses with a capsid of icosahedral symmetry and diameter of about 45 nm. Virions contain double-stranded DNA genomes approximately 5 kbp in length. Their protein coat is formed mainly by the VP1 protein, assembled into 72 pentamers [170]. Twelve VP1 pentamers are pentavalent, surrounded by five other ones; 60 pentamers are hexavalent, interacting with six neighboring pentamers. The minor capsid proteins VP2 or VP3 are attached to the central interior cavity of the VP1 pentamer. This interaction is provided by the C-terminus of the minor proteins, which forms an α-helix and has a hydrophobic character [171,172]. The C-terminus sequence is shared by both VP2 and VP3, since VP2 comprises the whole sequence of VP3 and has a unique N-terminal region approximately 120 amino acids long. Additionally, the N-terminal glycine of VP2 carries a myristyl moiety [173]. 

Mouse polyomavirus (MPyV) was the first polyomavirus discovered about mid of the last century [174]. Another polyomavirus that has attracted intensive attention and scientific interest is Simian Virus 40 (SV40). Human polyomaviruses have a high prevalence in the population. In terms of pathogenicity, the most important human polyomaviruses are JC, BK, and Merkel Cell Polyomavirus (MCPyV). While JC and BK viruses are apparently asymptomatic in healthy individuals, they can cause serious illnesses in the immune-compromised patients [175]. MCPyV is associated with rare but aggressive Merkel cell carcinomas [176].

#### 2.4.1. Cell Entry

Most polyomaviruses use sialic acids for initial attachment on the cell surface, a sugar-derived component that ends the extracellular part of larger molecules. However, polyomavirus receptors are, in general, species-specific. Some polyomaviruses use gangliosides as receptor molecules, e.g., mouse polyomavirus (MPyV) uses gangliosides GD1a and GT1b, SV40 binds to GM1, and BK virus interacts with GD1b and GT1b gangliosides [177,178,179]. 

Virus binding to the receptor has consequences for both interaction partners. As a result of SV40 binding to GM1, deep invaginations are formed in the cytoplasmic membrane [180]. Cavaldesi *et al.* showed [181] that treatment of viral-like particles (VLPs) of MPyV with sialic acid induces transition from the protease sensitive to the protease resistant form of all capsid proteins. This suggests that the MPyV capsid undergoes a conformational change after sialic acid binding which alters epitopes of the virus particle accessible to proteases. Furthermore, the receptor also influences further trafficking and fate of the viral particle inside the cell. For instance, the gangliosides were shown to stimulate MPyV trafficking to the productive pathway, while glycoproteins had the opposite role [178,182]. The correct targeting of the viral capsid is a probable prerequisite for membrane penetration and delivery of viral genomes into the nucleus (described in the next two sections below).

Following receptor binding, caveolin- and clathrin-independent endocytosis is used by many polyomaviruses to enter the cells. However, it is believed that JC internalization occurs via clathrin-coated pits [183,184,185,186,187].

#### 2.4.2. Trafficking Inside the Cell

The intracellular movement of polyomaviruses is characterized by several steps, which remain elusive even after decades of research. Virions have to reach ER for productive infection, but the route used to achieve this is not well understood. 

The MPyV receptor, GD1a, supports targeting of the virus to the ER [182]. Transport of virus to the ER, which is linked to productive infection, requires intact microtubules [188,189,190,191]. Interestingly, the low endosomal pH was shown to be crucial for MPyV infectivity [192]. Polyomaviruses were detected in early endosomes, endolysosomal compartments, in recycling endosomes and in ER [182,187,192,193]. Intriguingly, several research groups failed to detect polyomaviruses in the Golgi apparatus. This strongly suggests that polyomaviruses do not intersect this compartment [189,190,194]. Nevertheless, SV40 was found to use COP1 vesicles of the retrograde transport pathway that ensures transport from Golgi to the ER [195,196,197]. Similar to other viruses (for example, papillomaviruses), MPyV relies on dynein for the transport along microtubules as a part of productive infection [198]. 

#### 2.4.3. Roles of the Endoplasmic Reticulum

Delivery of polyomavirus particles to the ER appears to be important for subsequent steps of infection. Several groups have reported that polyomavirus particles are processed by ER resident enzymes [199,200,201]. Two categories of proteins involved in the polyomavirus infection are represented by the endoplasmic reticulum-associated degradation (ERAD) pathway components and chaperones. ERAD provides cytosolic translocation and degradation of misassembled proteins. SV40 infection and transport of the capsid proteins to the cytosol was demonstrated to use several enzymes that are associated with this pathway and misfolded protein processing [200,202,203]. Another polyomavirus, the mouse polyomavirus, requires activity of the Derlin-2 protein, which participates in the transfer of misfolded proteins from ER to the cytosol [204]. Recently, ERAD inhibitors were also shown to block BKV infection [205]. 

Protein disulfide isomerases (PDIs) also participate in polyomavirus and SV40 infection [199,200,201]. Since disulfide bonds are known to stabilize the capsid [206,207,208], it is plausible that alteration of these bonds can facilitate the particle disassembly. Indeed, Schelhaas *et al.* reported that virus treatment with PDI ERp57 leads to uncoupling of about five pentamers from the particle [200].

#### 2.4.4. Coat Determinants

The major coat protein, VP1, alone is sufficient to assemble into viral-like particles (VLPs) [209]. The minor capsid proteins, VP2 and VP3, are also dispensable for DNA encapsulation and particle assembly of MPyV and SV40 [210,211]. Nevertheless, VP2 was shown to enhance viral particle binding to the cell surface [210,212], but the minor proteins were not needed for internalization and ER targeting [202,213]. This is consistent with the observation that the GD1a receptor targets MPyV, as well as artificial particles coated by GD1a, to the ER [182]. The receptor binding is the competence of VP1 [214,215], supporting the theory of the major capsid protein being responsible and sufficient for viral delivery to the ER, the probable site of transmembrane escape (Section 2.4.3). 

The minor capsid proteins play a crucial role in the next step—ER escape and delivery of genomes to the nucleus [202,213]. Reported evidence suggested that the virus without VP2 or VP3 cannot escape to the cytosol [202,216], but viral particles containing VP1, VP2, VP3, and genome have been found in the cytoplasm [216]. This supports the model proposing that the virus travels through the cytosol and uses the nuclear pore complex to deliver the genome to the desired destination (discussed below). Interestingly, VP2 and VP3 also greatly increase efficiency of the gene transducing activity of polyomavirus-based vectors [212].

##### 2.4.4.1. Membrane Insertion Properties of the Minor Capsid Proteins

When expressed alone in the cells, the minor proteins can be observed (at least partially) in the cytoplasm associated with membranes [210,217,218]. In addition, *E. coli* producing SV40 VP3 died by cell lysis, because bacterial membranes were permeabilized upon the minor protein production [210]. In line with this finding, the expression of MPyV VP2 and VP3, fused at their C-terminus to EGFP, induced fast cell death of 3T3 cells, and immunoelectron microscopy revealed the minor protein association with damaged membranes of ER, nuclear envelope, and mitochondria [219].

Giorda *et al.* examined the pore formation by SV40 VP2 and VP3. This group confirmed the membrane lytic activity of the minor proteins by demonstrating that incubation of COS7 cells with purified VP2 or VP3 (produced as an N-terminal GST fusion protein) leads to cell lysis. Additionally, the membrane-perturbation ability of the polyomavirus minor capsid proteins was shown by experiments where the permeability to different polyethylene glycols was measured. The results strongly suggested that VP2 and VP3 create pores with inner diameter of approximately 3 nm and 4–6 nm, respectively [220]. VP2 and VP3 possess several hydrophobic domains, which can support oligomerization and serve as transmembrane segments, giving the ability to span the membrane several times.

Moreover, a recent study showed that purified SV40 late protein VP4 was able to fully integrate into the lipid bilayer of red blood cells. When the purified protein was applied to unilamellar vesicles, no flip-flop activity between the inner and outer membrane layer was detected [221]. This funding suggests that VP4 has a viroporin activity, can span the membrane bilayer, and create barrel pores. 

Giorda *et al.* [220] also investigated the hydrophobic regions of the minor proteins. Authors analyzed properties of the most probable hydrophobic segments of SV40 VP2 and VP3. As a result of both theoretical and practical approaches, three hydrophobic sequences revealed to be important: the polypeptides, labeled as HD1 (1–22 aa) and HD2 (70–100 aa), and located in the unique region of VP2, and HD6 (292–300 aa), which can be found in the C-terminus of both minor proteins. These domains exhibited the membrane insertion ability. This property was demonstrated by introduction of charged residues into the domains by single or double mutations, which diminished the membrane disruption activity of the purified full-length proteins. When the same mutations were introduced into the genome, viral assembly was not affected, but the infectivity was severely impaired [220].

Remarkably, the affinity of the HD6 segment of SV40 minor capsid proteins to membranes is in agreement with the studies done on the VP4 protein. This late protein seems to be unique for SV40 infection, and it is expressed at a very late stage of infection and contributes to cell lysis and to viral progeny release [222]. The VP4 translation starts from a downstream AUG start codon of VP3 transcript, and thus it represents the 125 C-terminal amino acids of VP2 and VP3. Consequently, it also includes the HD6 segment. As described above, VP4 was shown to perforate the membrane of liposomes and to form stable toroidal pores in the membrane [221]. 

The results concerning MPyV are not quite similar. Truncated VP3 of MPyV comprising the last 103 C-terminal amino acids (fused with EGFP) has lower membrane affinity and perforation activity than full-length EGFP fused minor proteins [219]. However, the peptide representing the last 35 amino acids of VP2 and VP3 shows the ability to fuse and disrupt liposomes in acidic, but not neutral pH environment (our unpublished results) [223]. It is possible that the very C-terminal segment of MPyV minor proteins has a conditional ability to bind membranes, which is influenced by the neighboring polypeptide properties and pH. In this way, the acidic environment and presence of several membrane spanning domains may well favor membrane insertion, while neutral pH and absence of sequences with membrane affinity in the truncated polypeptide result in weak protein insertion into the membranes.

In case of SV40 VP1 co-expression with VP2 and VP3, the minor proteins are inserted into pentamers, which make them unable to insert into and perforate membranes [210]. In eukaryotic cells, this complex translocates into the nucleus, where the particle assembly occurs [217,224]. This ensures production of the minor proteins needed for capsid formation without premature cell lysis. These evidences also allow us to speculate that the minor protein ability to disrupt lipid bilayers may be silent in the intact capsid, but is revealed (or triggered) after particle disassembly, which could lead to exposure or release of the minor proteins (see Section below).

These findings also suggest that the minor capsid proteins of at least MPyV and SV40 could use the membrane permeabilizing properties to disrupt the membrane, although the precise mechanism is not known. It is likely that these proteins are able to form oligomer pores, but they are too small to allow the viral genome translocation alone (models are discussed in the next section) (Table 3 and Table 4).

**Table 4:** Summary of the mechanisms used for membrane penetration by selected non-enveloped virus families.

Virus families	Membrane penetration mechanism	Conformation of the protein segment required for membrane penetration
**Parvoviridae**	Enzymatic activity	α-helices [109]
**Adenoviridae**	Protein-membrane interaction	Amphipathic α-helix [33]
**Papillomaviridae**		Transmembrane segment * [157,220,221]
**Polyomaviridae**		

* proposed mechanism according to recent publications, discussed in Section 2.3.3.1 and Section 2.4.4.1.

It should also be noted that while the minor proteins of SV40 and MPyV exhibit the ability to disrupt membranes, those of MCPyV seem not to have these properties, since they do not induce cell lysis when expressed in mammalian cells (our unpublished results) [225]. MCPyV and the related clade also seem to lack the VP3 protein in the virions. Thus, these species may use different mechanisms for vesicle escape than SV40 and MPyV [226].

##### 2.4.4.2. Role of the Minor Structural Proteins in the Virus Escape from ER

The survey of available evidence suggests that the minor structural proteins can be exposed after particle disassembly during virus trafficking, integrate into membranes, and promote virus translocation using their membrane perforation properties. Indeed, the minor proteins become detectable five hours post-infection of SV40, when most of the capsid protein signal is found in ER [196]. There, the virus undergoes at least partial disassembly [199,200]. As a result, this partial disassembly produces a hydrophobic particle that has affinity to the membrane [227] and exposes the SV40 VP2 N-terminus [202]. Likewise, the polypeptide comprising the first 29 amino acids of VP2 was shown to integrate into microsomes. 

In spite of this large number of experimental data, the way in which polyomaviruses cross the intracellular membranes remains under debate. One possibility is that minor proteins are exposed, while still attached to the capsid, and promote integration of the partially disassembled particle into the membrane bilayer. Their membrane-lytic activity could support the ER escape. Part of the minor protein population may be released to form pores in the membranes, but these pores are too small to allow the viral genome translocation. Free minor proteins integrated into the membrane bilayer could also locally destabilize the membrane, which would facilitate translocation of the viral particle. 

One model asserts the ERAD pathway to assist transmembrane translocation. How ERAD could handle such a large particle, as was observed by Inoue and Tsai [228], in the cytoplasm remains a question. The pore created by ERAD could possibly expand thanks to the membrane destabilization induced by the minor proteins. The minor capsid proteins may accompany the genome to the cytoplasm and to the nucleus.

Moreover, new evidence has emerged recently to support the ER-to-cytosol translocation model. Walczak *et al.* (2014) [229] have identified potential cytosolic interaction partners for this step. The cytosolic chaperone SGTA (small glutamine-rich tetratricopeptide repeat-containing protein α) was shown to bind the SV40 particle and to be required for its transport from the ER membrane into the cytosol. In addition, the two J-proteins, DnaJB14 and DnaJB12, which were previously shown to assist the SV40 membrane penetration, are also implicated in this process mediated by SGTA [216,229].

On the other hand, it was reported that the requirement of ERAD pathway for cytosolic entry varied between SV40 and BK, and between immortalized CV-1 cells and primary renal proximal tubule epithelial cells (RPTE cells) [205]. As well, cytosolic translocation of the VP1 protein did not correlate with the appearance of viral genomes, and inhibition of ERAD did not prevent BK VP1 detection there, which is not consistent with the observation of viral particles containing all capsid proteins and the genome in the cytoplasm. One of the possible explanations could be that the composition of the genome-capsid complex exported to the cytoplasm differs between SV40 and BKV. For example, one can speculate that the BK genome leaves ER accompanied by VP2 and VP3 only, while the SV40 genome can be associated with all capsid proteins. Another option is that particles containing all capsid proteins do not undergo productive infection.

In general, discrimination between the productive and nonproductive route of viral infection represents a great challenge for researchers. It is reasonable to assume that if a defective viral particle reaches ER, the cell has to deal with it. One should not ignore the possibility that the ER-to-cytosol translocation pathway is a route to degradation and, perhaps, is not associated with productive infection at all. Many chaperones and PDI knockdown assays have been proposed in order to prove this model. However, these experimental approaches are likely to affect a vast array of cellular proteins and, therefore, some of the observed effects on viral infection can be indirect.

Alternatively, the minor capsid proteins could assist the nuclear envelope destabilization and viral translocation directly from ER to the nucleus (see next Section).

#### 2.4.5. Post-Vesicular Steps

Hypothetically, two main options exist for the ER resident virus to reach the nucleus—via escape to the cytoplasm or fusion/penetration directly through the nuclear envelope. Unfortunately, our current knowledge does not allow us to ultimately exclude either model for polyomavirus infection.

The model where the cytoplasm as a compartment is included in the productive infection is supported by experiments of Nakanishi *et al.*, 1996, which show that introduction of anti-VP1 or anti-VP3 antibodies into the cytoplasm prevents infection. Further experiments also revealed that SV40 DNA interacts with importins α and β and that viral genomic DNA exists in a complex with VP1 and the minor protein, VP3. Importantly, the minor protein VP3 was shown to be indispensable for this interaction with importins. In addition, the nuclear localization signal of VP3 was found to be essential for recognition by importin α2/β and genome delivery into the nucleus [213,230,231]. These data also suggest that the viral genome, associated with VP2/VP3, enters the nucleus from the cytoplasm. However, Kuksin and Norkin [232] failed to detect VP2 or VP3 in the nucleus of infected cells. Their results advocated that the genome enters the nucleus free of VP2 and VP3. 

As mentioned above, another model proposes viral escape directly to the nucleus. Early electron microscopy studies found viral particles in the nucleus, together with nuclear envelope disruptions in the viral particle proximity [233,234]. These observations suggested viral entry through the nuclear membrane perforation, because particles of this size are too large for the translocation through the nuclear pore complex. More recently, Butin-Israeli and co-authors [235] corroborated this model by demonstrating alterations of the shape and lamin composition of SV40-infected cells. The time when the deformation of nuclear envelope was detected overlapped with that of the entry of viral genomes to the nucleus. Interestingly, the VP1 pentamer was sufficient to induce the signals leading to fluctuations in lamin A/C levels. This alternative theory interprets the appearance of the capsid proteins in the cytosol as part of the degradative pathway and defense mechanism of the cell, which would be consistent with the fact that the majority of viral particles are unable to establish productive infection.

## 3. Conclusions

In this review, we intended to analyze the current knowledge of the role of capsid proteins in the transmembrane translocation of small non-enveloped DNA viruses during early stages of infection. In general, this step of infection seems to be inefficient and often rate limiting during intracellular trafficking, since many viral particles can be internalized but just a few of them reach the nucleus. Table 3 serves to point out the most important features of the membrane penetration capsid protein (MPCP). To start with, MPCP constitutes rather a minor part of the capsid and its major part is not accessible from the particle exterior. The membrane lytic properties need to be spatially and temporally regulated, in the same fashion as an assassin uses a dagger. The virus needs to be delivered to a specific cellular compartment, where the MCPC action takes place. Therefore, MPCP is hidden inside the capsid and is released or exposed after induction. The signals leading to MPCP exposure are tightly related to changes of the capsid during virus trafficking. Such changes are triggered, for example, by interaction with the receptors or capsid disassembly. In the case of polyomavirus, the induction of MPCP appears to be more specific. Minor capsid proteins are exposed after treatment with ER resident enzymes, which are specific for the membrane exit compartment, but not for others that the particle traverses.

The role of adenoviral protein VI in infection appears to end up with the membrane penetration step. The MPCPs of parvo-, papilloma-, and (possibly) polyomaviruses stay linked with the capsid and/or viral genome. Evidences exist that papillomavirus and polyomavirus MPCP remain associated with the genome until it reaches the nucleus.

Table 3 and Table 4 summarize the membrane penetration mechanisms and compare their main properties and players. Among viral families discussed in this review, parvoviruses reach the cytosol in a distinct manner that involves enzymatic digestion of lipids. Unlike parvoviruses, the MPCPs of other viral families have membrane insertion properties. For instance, adenoviral protein VI amphipathic helix leads to the membrane rupture. In the case of papillomavirus and polyomavirus, the mechanism of membrane penetration has not been firmly established, but it is likely to be related to the transmembrane segment of MCPCs, which tends to oligomerize. MCPCs can promote membrane destabilization and/or insertion of the viral particle to the membrane. 

We hope that our review will create a complex view concerning this topic. In fact, significant progress has been made in this field, including identification of capsid proteins mediating transmembrane translocation and analysis of their domains and residues responsible for this function (Table 3 and Table 4). The involvement of other capsid proteins has also been described, as well as the role of some of the cellular proteins and processes. Despite that, the elucidation of the precise mechanism of transmembrane translocation remains a challenge even after decades of research. Nevertheless, several models have been proposed, which are more or less supported by experimental data, but further studies are needed to fully understand this critical step of infection. 
